# Nanoscale Phase Evolution during Continuum Decomposition of Fe-Cr Alloys

**DOI:** 10.3390/ma10121431

**Published:** 2017-12-15

**Authors:** Yongsheng Li, Lihui Zhu, Chengwei Liu, Shujing Shi

**Affiliations:** School of Materials Science and Engineering, Nanjing University of Science and Technology, Nanjing 210094, China; zlhqjulie@163.com (L.Z.); cwliu16@163.com (C.L.); ssj3677@163.com (S.S.)

**Keywords:** phase decomposition, Fe-Cr alloys, phase-field

## Abstract

The continuum decomposition of the Fe-Cr alloys from initial phase separation to steady-state coarsening with concentrations varying from 25 at % Cr and 30 at % Cr to 33 at % Cr aged at 750 K was studied by utilizing three-dimensional phase-field simulations. The dynamic stages of separation of nanoscale Cr-enriched α′ phase were distinguished by the evolution of the volume fraction, particle number density and the average particle radius of the α′ phase. The stage of steady-state coarsening was characterized with an equilibrium volume fraction and decreasing particle number density. The coarsening rate constant by linear fitting of the cube of average radius and aging time shows an increase with the increasing Cr concentration. The time exponents decrease from the growth and coarsening stage to the steady-state coarsening stage and show a dependence on the particles number density at different concentrations. The quantitative evolutions of α′ phase via nucleation growth and spinodal decomposition are theoretically helpful for understanding the microstructure evolution with aging time in Fe-Cr alloys.

## 1. Introduction

Fe-Cr alloys, the basic component of duplex stainless-steel (DSS), have exhibited excellent mechanical properties at high temperatures. As a structural material, high-Cr DSS has been used in nuclear power plants [1,2,3]. The excellent combination of mechanical properties and corrosion resistance of DSS is obtained from the balanced amount of ferrite and austenite in the microstructure. However, Fe-Cr alloys are susceptible to embrittlement at aging temperatures ranging from 300–500 °C [4,5,6] or under radiation exposure [7]. This embrittlement is attributed to the spinodal decomposition of the solid state into an ultrafine mixture of Cr-enriched and Fe-enriched phases. The separation of the Cr-enriched phase occurs rapidly at the early stages of aging, which has been demonstrated by concentration wavelength and hardness variations of Fe-Cr alloys [8]. The composition is a dominant factor for the decomposition kinetics [9]. Therefore, the phase separation dynamics in Fe-Cr alloys are potentially useful for predication of the morphology evolution and property change, and much attention has been focused on this question [10,11,12,13,14,15].

The separation of the Cr-enriched phase also can occur via the nucleation and growth in Fe-Cr alloys with low Cr concentration [16,17]. The Mössbauer spectroscopy also demonstrated the decomposition via nucleation and growth in Fe-24 at % Cr alloy at 475 °C [18]. Atom probe tomography (APT) results showed that the α′ phase separation is by means of non-classical nucleation and growth in Fe-20 at % Cr alloy aged at 773 K from 50–1067 h [3]. A transient coarsening regime was observed for the overlap of the nucleation, growth and coarsening, and the steady-state coarsening was not observed for an aging time of 1067 h, while a linear evolution of the cube of the mean particle radius was presented. The Ising model demonstrates that there was a gradual transition from nucleation and growth to spinodal decomposition at the spinodal line [19], while a sharp change was predicted by the Cahn-Hilliard theory of spinodal decomposition [20]. Xiong et al. [21] summarized the decomposition mechanisms of the Fe-Cr alloys for the experimental and theoretical results, which show overlapped regions for the nucleation growth and spinodal decomposition. In addition, their three dimensional atom probe tomograph (3D-APT) results showed that a transition region from nucleation and growth to spinodal decomposition exists in the composition regions from 24–36.3 at % Cr [22]. Therefore, the transition mechanism from nucleation and growth to spinodal decomposition in Fe-Cr alloys is theoretically interesting and practicably important.

In addition, the dynamics of growth and coarsening of the α′ phase is also indispensable for quantitative analysis and morphology prediction during aging. An atomic-scale analysis of phase decomposition in a thermally aged Fe-25 at % Cr alloy at 500 °C using a 3DAP and atomistic kinetics Monte Carlo (AKMC) simulation was presented, in which the fitting of the length scale and time, *L*^3^~*t* and *L*~*t*^1/3^ both presented a linear relationship [23]. Miller et al. [24,25,26] studied the kinetics of early stage phase decomposition and the spinodal morphology in Fe-Cr alloys. The microstructure scale simulated by the Cahn-Hilliard-Cook equation showed a time exponent close to the value of 1/3 of that predicted by Lifshitz-Slyozov-Wagner (LSW) [27,28] theory for the coarsening of isolated precipitates. However, their experimental fitting yielded a time exponent of 0.25 ± 0.03, and the Monte Carlo simulation fitted a power law relationship with a time exponent of 0.21 ± 0.03 [25]. Rogers et al. [29] used finite difference methods to study a 2D percolating spinodal system with a continuous order parameter, and found that the domains coarsen with a time exponent of 1/3 at a late time independent of thermal noise. The separation of the α′ phase by spinodal decomposition was studied in a Fe-42 at % Cr alloy at 700 K, 725 K and 750 K, the results showed an increased growth and coarsening rate with the aging temperature increases [30].

However, the continuum dynamics of α′ phase from initial separation to growth and coarsening via nucleation and growth to spinodal decomposition with Cr concentration increasing need a theoretical clarification in the Fe-Cr alloys. In this work, the alloys inside the regions of nucleation and growth, near the spinodal line and inside the spinodal region, were chosen to ensure phase separation from nucleation and growth to spinodal decomposition. The early stage evolving for the minute nano-scale particles in the Fe-Cr system is a notable challenge [10], so we utilized a three dimensional (3D) phase-field model [31,32] to investigate the evolution of the morphology, the time exponent of the length scale of steady-state coarsening and the particle number density of α′ phase in Fe-25 at % Cr, Fe-30 at % Cr and Fe-33 at % Cr alloys aged at 750 K. The stages of phase separation from the initial separation to growth and steady-state coarsening were distinguished by the temporal variation of the particle number density, the volume fraction and the average radius of α′ phase.

## 2. Phase-Field Model and Calculation Methods

### 2.1. Phase-Field Model

The composition evolution in Fe-Cr alloys can be described by the Cahn-Hilliard diffusion equation [33]

(1) $$\frac{\partial c(r,t)}{\partial t}=V_{m}\nabla \cdot \left[M\nabla \left(\frac{\delta F}{\delta c(r,t)}\right)\right]$$

where *c* is the nominal composition of Cr, *M* is the chemical mobility given by Darken’s equation [34,35] $M=\frac{1}{V_{m}}\left[cM_{\mathrm{Fe}}+(1-c)M_{\mathrm{Cr}}\right]c(1-c)$, where $M_{\mathrm{Fe}}$ and $M_{\mathrm{Cr}}$ are the atomic mobility of Fe and Cr, respectively, which are related to the diffusivity through Einstein’s relation $M_{i}=D_{i}/RT$, where *i* denotes the element Fe or Cr, *D_i_* is the diffusion coefficient, and the diffusion constant for Fe and Cr are 1.2 × 10^−4^ and 2.0 × 10^−5^, respectively, the activation energy is 294 kJ·mol^−1^ for Fe and 308 kJ·mol^−1^ for Cr [36,37]. Recently, the mobility matrix related with the local composition was utilized in the phase-field model for multi-component alloys [38], while the calculation is more complex than the linear variation between the elements.

The total free energy *F* of Fe-Cr alloys includes the chemical free energy, interfacial energy and elastic strain energy induced by the composition inhomogeneity between the α and α′ phase, and can be expressed as [39]:

(2) $$F=\int_{V}\left\{\frac{1}{V_{m}}\left[G+\frac{1}{2}\kappa {\left(\nabla c\right)}^{2}\right]+E_{el}\right\}dV$$

where $V_{m}$ is the molar volume of the alloy, κ is the gradient energy coefficient, $E_{el}$ is the elastic energy density per unit volume, and *G* is the molar Gibbs free energy given by [40]

(3) $$G=\left(1-c\right)G_{\mathrm{Fe}}^{0}+cG_{\mathrm{Cr}}^{0}+L_{\mathrm{FeCr}}c\left(1-c\right)+RT\left[c\ln c+(1-c)\ln(1-c)\right]+G_{m}$$

where $G_{\mathrm{Fe}}^{0}$ and $G_{\mathrm{Cr}}^{0}$ are the energies of the pure elements [41], *R* is the gas constant, *T* is the absolute temperature, $L_{\mathrm{FeCr}}$ is the interaction parameter between Fe and Cr, and is adopted as $L_{\mathrm{FeCr}}=20,500-9.68T$ (J·mol^−1^) [42], $G_{m}$ is the magnetic ordering contribution to the Gibbs free energy, and its expression can refer to the literature [15,40].

The concentration gradient coefficient for the nearest neighbor interactions is expressed by $\kappa =\frac{1}{6}r_{0}^{2}L_{\mathrm{FeCr}}$ [36], where $r_{0}$ is the interatomic distance at a stress-free state and changes with composition obey the Vergard’s law. It should be noted that the composition effects are neglected in the gradient coefficient, which may have an influence on the interface width [43,44].

**Figure 1:**
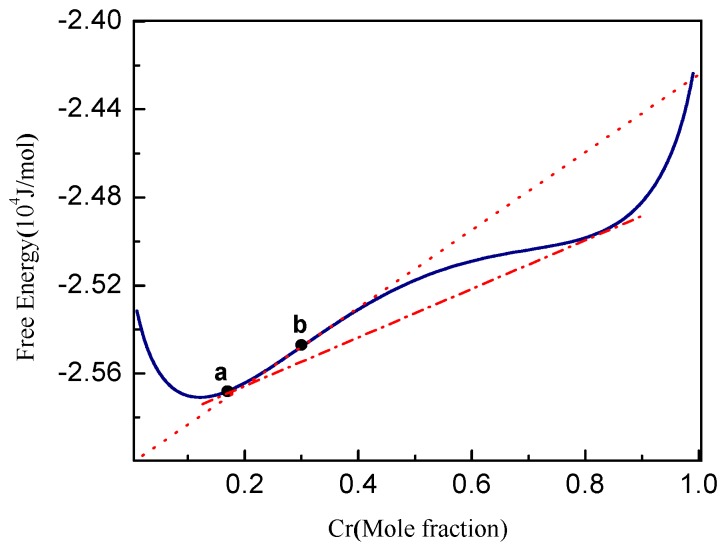
Free energy of the Fe-Cr alloy at 750 K, point **a** with Cr concentration *c*_Cr_ = 0.17 delegates the boundary composition of phase separation, point **b** (*c*_Cr_ = 0.30) delegates the critical composition of spinodal decomposition. The dotted line is the tangent through the spinodal boundary and dash-dotted line is the tangent of equilibrium composition.

The free energy of Fe-Cr alloy including the Gibbs free energy *G* and elastic strain energy at 750 K is plotted in Figure 1, where the compositions for phase separation and spinodal decomposition are labelled in the curve, and points **a** and **b** are the composition boundary for phase separation and spinodal decomposition at 750 K, respectively. The elastic strain energy density per unit volume can be calculated by $E_{el}=\frac{1}{2}C_{ijkl}\varepsilon_{ij}^{\mathrm{el}}\varepsilon_{kl}^{\mathrm{el}}$. For the detailed solution for elastic strain $\varepsilon_{ij}^{\mathrm{el}}$, refer to the literature [13,45,46].

### 2.2. Numerical Calculation

By substituting Equation (2) into Equation (1) and performing the dimensionless, we can calculate numerically by using the semi-implicit Fourier spectrum method [47,48] with a time step of $\Delta t^{*}=0.001$. *T*_c_ = 900 K [21,24] is the critical temperature of spinodal decomposition of the alloy. The lattice parameters are $a_{\mathrm{Fe}}=0.2866\;\mathrm{nm}$ and $a_{\mathrm{Cr}}=0.2882\;\mathrm{nm}$ [49], the dimensionless grid size is chosen as $\Delta x^{*}=\Delta y^{*}=\Delta z^{*}=1.0$, and the simulation cell size is 128$\Delta x^{*}$ × 128$\Delta y^{*}$ × 128$\Delta z^{*}$. The elastic constants of Fe are approximately chosen as $C_{11}^{\mathrm{Fe}}$ = 197, $C_{12}^{\mathrm{Fe}}$ = 128 and $C_{44}^{\mathrm{Fe}}$ = 107 GPa at 773 K [50]; those for elemental Cr are $C_{11}^{\mathrm{Cr}}$ = 358, $C_{12}^{\mathrm{Cr}}$ = 106 and $C_{44}^{\mathrm{Cr}}$ = 95 GPa at 650 K [51]. The chemical mobility *M* is updated for each calculation step with the iterative temporal changes in composition throughout the simulation.

A random thermal fluctuation (Langevin noise) at a magnitude of [−0.002, 0.002] is introduced into the initial composition in the simulation to trigger the phase separation, and an iteration of the thermal fluctuation is performed for the alloys in the metastable regions (between point **a** and **b** of Figure 1) of nucleation and growth (Fe-25 at % Cr) or near the boundary of spinodal decomposition (Fe-30 at % Cr). It should be noted that the thermal fluctuation should have a minimum magnitude for triggering the phase decomposition, as a large fluctuation may affect the initial particle number and the particles radius. As we know, there are different approaches to simulate the nucleation in the phase field simulation, such as the Langevin noise method and the explicit nucleation method [52,53,54]. The other numerical algorithms for nucleation problems include computing saddle points and minimum energy path [54]. The merits and drawbacks of these methods are discussed in the literature [52,53,54], in addition, the implementation of these new approaches is not straight forward and is computationally complex.

## 3. Results and Discussion

### 3.1. Phase Separation in Metastable Regions

**Figure 2:**
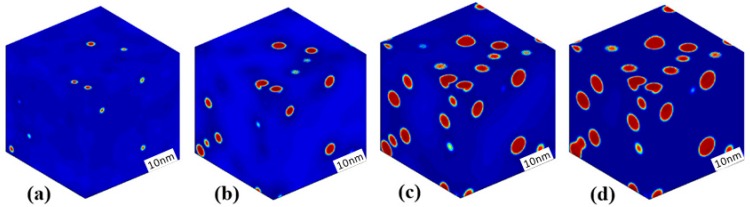
3D morphology evolution of the Cr-enriched α′ phase in the Fe-25 at % Cr alloy aged at 750 K, (**a**) *t* = 46 h; (**b**) *t* = 115 h; (**c**) *t* = 5390 h; (**d**) *t* = 6773 h.

The morphology evolution of the Cr-enriched α′ phase in the Fe-25 at % Cr alloy aged at 750 K is presented in Figure 2, where the red regions depict the Cr-enriched α′ phase and the blue regions depict the Fe-enriched α phase. For the alloy locates in the metastable region, the initial thermal fluctuation was added for an iteration of 400 time steps, and was removed when the system was able to develop automatically. It can be seen from Figure 2a,b that some of the initially emerging Cr-enriched clusters develop into the α′ phase as the aging progresses. There are deep blue regions of low Cr concentration around the α′ phase, which is caused by the Cr atom diffusing from the matrix α to support the growth of α′ phase. Then, the α′ phase precipitates gradually from the matrix and its number increases, as shown in Figure 2b,c. The following coarsening of the spherical α′ phase occurs through individual coarsening for the separated particles and coalescence coarsening for some neighbor particles, as shown in Figure 2d. 

The spherical α′ particles in the Fe-25 at % Cr alloy aged at 750 K have an average radius of approximately 3.4 nm after aging for 6773 h. The atom probe tomography (APT) result for the α′ radius is approximately 2.53 ± 0.51 nm in Fe-20 at % Cr alloy aged at 773 K for 1067 h [3]. The characteristic length of α′ is 3 nm in a thermally aged Fe-25 at % Cr alloy at 500 °C for 240 h detected by the 3D atom probe (3DAP) [23]. Lopez-Hirata et al. studied the phase decomposition of a Fe-40 at % Cr alloy aged at 475 °C and 500 °C [55], and their results revealed a Cr-enriched phase with a size of less than 10 nm. The radius of the α′ phase is approximately 4 nm in Fe-40 at % Cr alloy aged at 773 K for 500–750 h detected by a TEM experiment [8]. Thus, the simulated morphologies of α′ phase are consistent with the previous experimental results.

**Figure 3:**
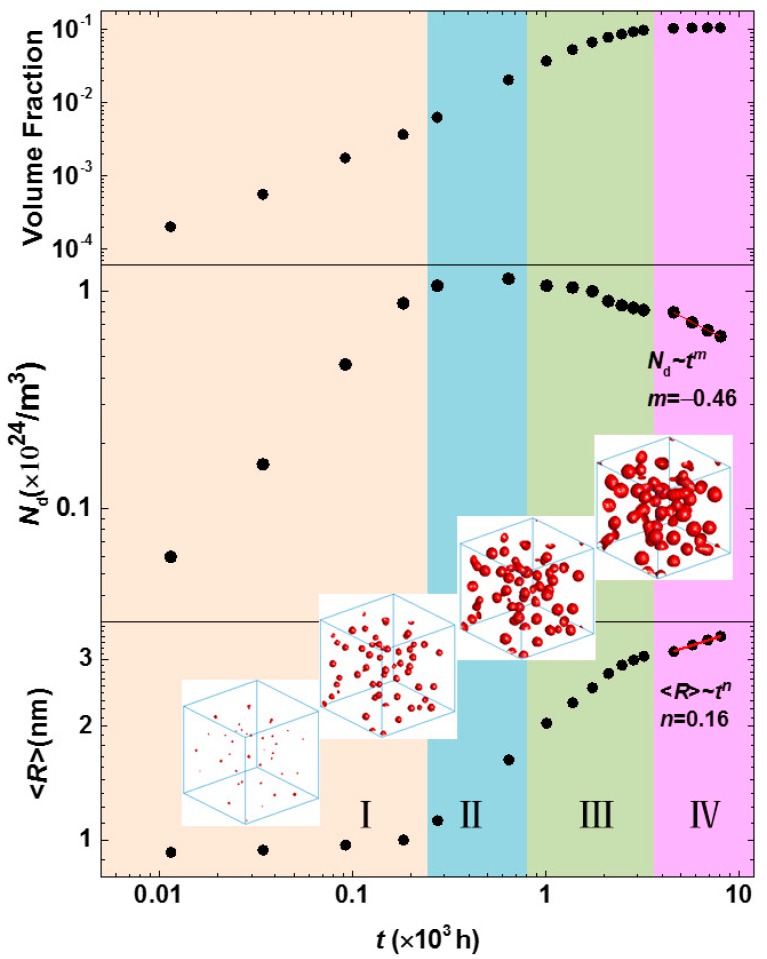
The temporal evolution of the volume fraction, particle number density and the average particle radius of the Cr-enriched α′ phase in the Fe-25 at % Cr alloy aged at 750 K. The inserted morphology evolution is for 3D α′ particles from initial growth to coarsening.

The separation dynamics of the α′ phase in Fe-25 at % Cr alloy aged at 750 K were analyzed by the temporal variation of volume fraction, particle number density and the average particle radius of the α′ phase. The particle number density *N*_d_ is defined by the α′ phase number per unit volume, and the average particle radius <*R*> is an average radius of an approximate spherical shape with a volume *V* of the α′ phase, <*R*> = $\frac{1}{N_{p}}\sum_{i=1}^{N_{p}}{\left(3V/4\pi \right)}^{1/3}$, where *N*_p_ is the total number of the α′ phases. As shown in Figure 3, the separation of the α′ phase can be separated into four stages: (I) nucleation of the α′ phase at the initial phase separation; (II) nucleation and growth; (III) concurrent growth and coarsening once the maximum value of *N*_d_ is achieved; (IV) steady-state coarsening with a stable volume fraction. The time exponent at the steady-state coarsening is calculated by fitting the relationships of *N*_d_~*t*^m^ and the <*R*>~*t^n^*, they are *m* = −0.46 and *n* = 0.16.

**Figure 4:**
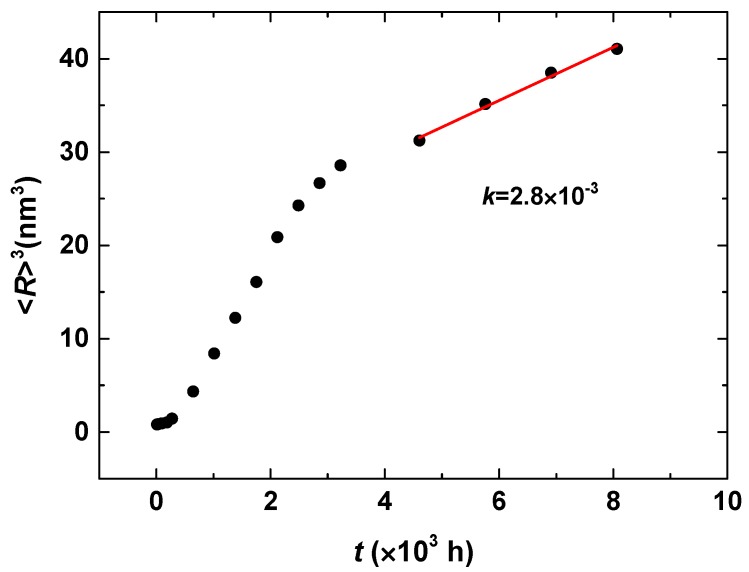
The temporal evolution of the cube of the average particle radius of the Cr-enriched α′ phase at steady-state coarsening in the Fe-25 at % Cr.

The time exponent 0.16 of the steady-state coarsening is less than 1/3 from classical LSW theory [26,27]. However, the LSW theory is suitable under the conditions of dilute solutions with near-zero volume fractions when there are no elastic interactions between precipitates. It is implicit that the evaporation-condensation mechanism, i.e., Ostwald ripening, is operative in LSW theory. In addition, the fitted exponent in the steady-state coarsening has a decrease in the early growth and coarsening stage with the exponent 0.41, and the limited particles number also affects the statistic. Miller also found a small time exponent 0.25 ± 0.03 by fitting their experimental results [25]. Pareige did not fit the time exponent when studying the precipitation of the α′ phase in Fe-Cr alloys with 3DAP; however, they demonstrated a linear relationship between the domain scale *L* and time *t*, *L*^3^~*t* and *L*~*t*^1/3^ were both presented [23]. The cube of the average particle size <*R*>^3^ and time *t* were fitted at the steady-state coarsening stage, as shown in Figure 4, which also shows a linear relationship with the coarsening rate constant *k* = 2.8 × 10^−3^ independent of the time exponent of *n* = 0.16. Therefore, the linear fitting of <*R*>^3^~*t* does not imply the time exponent *n* = 1/3. A time exponent *n* deduced from the average radius and time <*R*>~*t^n^* indicates the dynamics of phase precipitation, which also showed in the nickel-based alloys [56].

### 3.2. Phase Separation Near the Spinodal Boundary

**Figure 5:**
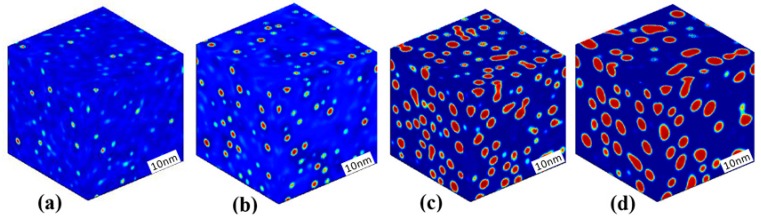
3D morphology of the Cr-enriched α′ phase in the Fe-30 at % Cr alloy aged at 750 K, (**a**) *t* = 70 h; (**b**) *t* = 138 h; (**c**) *t* = 691 h; (**d**) *t* = 2995 h.

Figure 5 displays the morphology evolution of the α′ phase in the Fe-30 at % Cr alloy aged at 750 K, which is near the point b of Figure 1. In this analysis, the magnitude of initial thermal fluctuation is [−0.002, 0.002], and the length of time steps is the same as those of Figure 2, but the number of iterations of thermal fluctuation is reduced to 100 time steps. The iterative addition of thermal fluctuation for the precipitation indicates that nucleation and growth still happen for alloys near the spinodal line. The initial morphology of the α′ phases shown in Figure 5a is similar to that of Figure 2a, where the Cr-enriched particles emerge separately. Then, the spherical α′ phases grow and coarsen continuously, as shown in Figure 5b–d, in which the coalescence coarsening and Ostwald ripening are both present and some neighboring α′ particles interconnect each other and form a worm shape. As the coarsening progresses, the distance between the particles is enlarged, the diffusion distance of Cr to the α′ phase increases, and the Ostwald ripening becomes more obvious, as shown in Figure 5c,d. Therefore, the coarsening of the α′ phase in the Fe-30 at % Cr alloy aged at 750 K prefers the coalescence coarsening at initial stages but is dominated by the Ostwald ripening at later stages.

**Figure 6:**
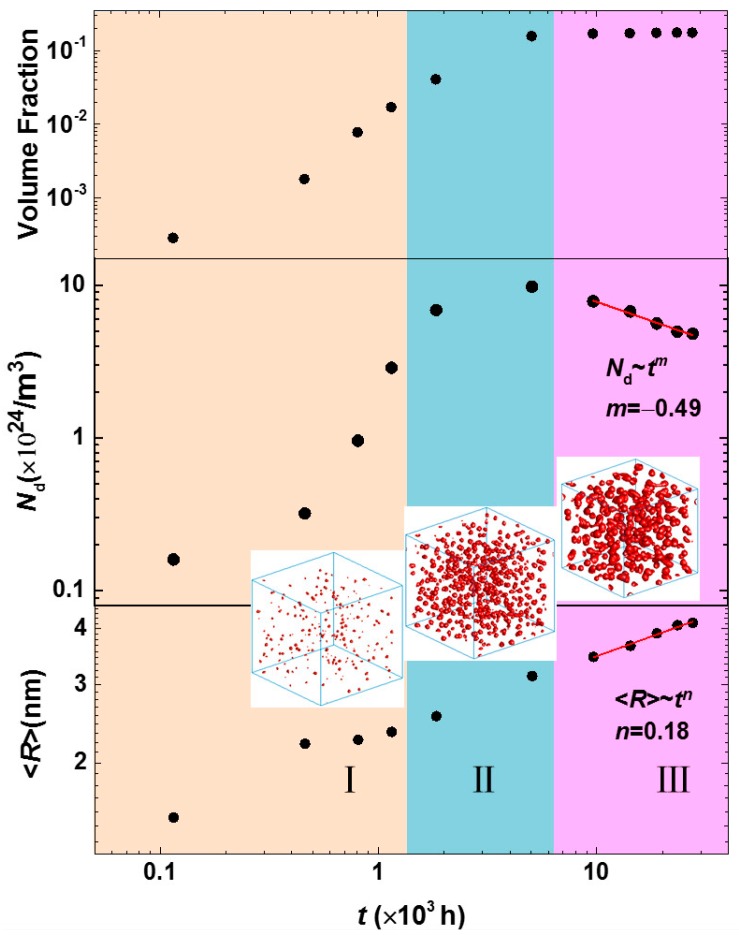
The temporal evolution of the volume fraction, particle number density and the average particle radius of the Cr-enriched α′ phase in the Fe-30 at % Cr alloy aged at 750 K.

Figure 6 shows the temporal dynamics evolution of the volume fraction, particle number density and the average particle radius of the α′ phase. The dynamics of phase separation in Fe-30 at % Cr alloy aged at 750 K show three stages: (I) fast precipitation and concurrent growth of the α′ phase with an increased particle number, volume fraction and radius; (II) slow precipitation and growth before the maximum *N*_d_ is achieved; and (III) steady-state coarsening, in which the volume fraction has a steady value. The dynamics exponents for the particle number density *N*_d_~*t*^m^ is *m* = −0.49 and for the average particle radius <*R*>~*t^n^* is *n* = 0.18. The magnitudes of the time exponents are close to those of Fe-25 at % Cr alloy aged at 750 K. The cube of the average particle radius <*R*>^3^ and time *t* was also fitted with a linear function for the Fe-30 at % Cr alloys aged at 750 K at the steady-state coarsening stage, the coarsening rate constant is *k* = 1.7 × 10^−2^. The variation of coarsening rate constants indicates the increased coarsening rate as the Cr concentration increases, as shown in Table 1.

### 3.3. Phase Separation in Spinodal Decomposition Region

**Figure 7:**
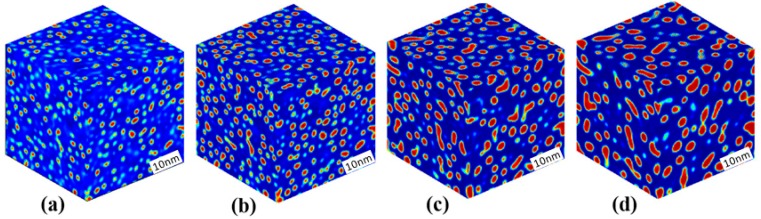
3D morphology of the Cr-enriched α′ phase in the Fe-33 at % Cr alloy aged at 750 K, (**a**) *t* = 70 h; (**b**) *t* = 138 h; (**c**) *t* = 506 h; (**d**) *t* = 1152 h.

Figure 7 displays the morphology evolution of the α′ phase in the Fe-33 at % Cr alloy aged at 750 K, where more α′ particles precipitate, as shown in Figure 7a, than in any of the alloys in or near the regions of nucleation and growth. For this alloy, the magnitude of initial thermal fluctuation is the same as Fe-30 at % Cr alloy aged at 750 K while without iterations. As the precipitation and growth of the α′ particles progresses, the connection of neighbor particles progresses simultaneously, as shown in Figure 7a,b. Then, Ostwald ripening accompanied by coalescence coarsening occurs in Figure 7c,d as the particle distance increases. Therefore, the coarsening of the α′ phase in the Fe-33 at % Cr alloy aged at 750 K is dominated by the coalescence coarsening of the initial stages followed by concurrent coalescence coarsening and Ostwald ripening at later stages. Due to the coalescence of the α′ particles, a worm-shaped α′ phase is present at the aging time *t* = 1152 h.

The time exponents were also calculated by fitting the particle number density and the average particle radius of steady-state coarsening of the Cr-enriched α′ phase in the Fe-33 at % Cr alloy aged at 750 K. Table 1 shows the time exponents of the average radius and particle number density of the α′ phase with different compositions. The time exponents for the average radius are less than the 1/3 expected from classical LSW theory at 750 K. The coarsening time exponent increases as the Cr concentration increases from 25 at % to 33 at % at 750 K. The present simulation includes the elastic interactions between the α and α′ solid phases with a large volume fraction. In addition, the higher particle number density has a smaller particle distance for the high concentration or low temperature aging, which favors the coalescence coarsening and Ostwald ripening via short-distance diffusion. Therefore, the coarsening time exponents are increased as the particle number density changes from 1.13 × 10^24^ to 9.7 × 10^24^ and 2.5 × 10^25^ m^−3^ as the Cr concentration increases at 750 K. As a result, the coarsening time exponent of the α′ phase depends on the composition that relates with the particle number density.

Additionally, there are some experimental results that show the coarsening time exponent is less than 1/3 [29,57,58,59]. The autocorrelation function from the energy compensated atomic probe in Hyde’s results also showed a time exponent of ~0.21, and the PoSAP (position-sensitive atom probe) time exponent was ~0.25 in Fe-30 at % Cr alloy at 773 K [25]. While Hyde’s numerical results simulated by the Cahn-Hilliard-Cook equation follow a power law close to the 1/3 of the classical LSW theory, it should be noted that the elastic energy and magnetic energy are not considered in the Cahn-Hilliard-Cook equation [24].

As a result of the phase separation in the Fe-Cr alloys from nucleation and growth to spinodal decomposition, the coarsening time exponents of steady-state coarsening deduced by the <*R*>~*t^n^* show values from 0.16 to 0.18 as the concentration changed from 0.25 to 33 at % Cr aging at 750 K. The high concentration alloy with 35 at % Cr aging at 750 K has a time exponent 0.36 at 750 K, the temperature dependent exponent is also shown in the Fe-35 at % Cr alloy [60]. The coarsening rate constant *k* deduced from <*R*>^3^~*kt* increases with the concentration, which is attributed to the short distances of diffusion required at high concentrations. The statistic of simulation may include some shortcomings, such as the composition independent interface energy and the simplified mobility, which has an effect on the quantitative calculation of the coarsening time exponent. Therefore, further work using the abundant thermodynamic database and the diffusion coefficients is expected for the quantitative simulation.

## 4. Conclusions

The continuum separation dynamics and the morphology of nanoscale Cr-enriched α′ phase in Fe-Cr alloys aged at 750 K were quantitatively investigated using three-dimensional phase-field simulations. The dynamic stages from the initial separation and growth to steady-state coarsening of α′ phase were distinguished by the temporal evolution of the volume fraction, particle number density and the average particle radius of the α′ phase, accompanied by the phase separation mechanisms of nucleation growth and spinodal decomposition deduced from the free energy curve.

The α′ phase proceeds through the stages of nucleation, nucleation and growth, growth and coarsening and steady-state coarsening for the mechanism of nucleation and growth. The phase decomposition is accelerated as the Cr concentration is increased. The time exponents of steady-state coarsening show an increase from 0.16 of Fe-25 at % Cr aged at 750 K (nucleation and growth) to 0.18 of Fe-33 at % Cr aged at 750 K (spinodal decomposition) and as the Cr concentration increases, the large particles number density results in the increased time exponent. Simultaneously, the fitting of the cube of average radius and time, <*R*>^3^~*t*, shows an increased coarsening rate constant with the alloy concentration increasing, as a result of the short distances of diffusion for high Cr concentrations alloy. The results for the continuum dynamics evolution are scientific and applicable to phase separation occurring via nucleation and growth and spinodal decomposition with variable compositions in the Fe-Cr alloys.

## Figures and Tables

**Table 1:** Coarsening rate constants *k* and time exponents for the average radius and particle number density of the α′ phase in Fe-Cr alloys aged at 750 K.

Cr (at %)	25	30	33
*k*	2.8 × 10^−3^	1.7 × 10^−2^	2.1 × 10^−2^
*n*	0.16	0.18	0.18
*m*	−0.46	−0.49	−0.48
